# Biomechanical Evaluation of a Novel V-Shaped A2 Pulley Reconstruction Technique Using a Free Palmaris Longus Tendon Graft Tenodesis

**DOI:** 10.3390/jcm14041092

**Published:** 2025-02-08

**Authors:** Gabriel Halát, Hannah E. Halát, Lukas L. Negrin, Thomas Koch, Lena Hirtler, Christoph Fuchssteiner, Stefan Hajdu

**Affiliations:** 1 Department of Orthopedics and Trauma Surgery, Medical University of Vienna, Währinger Gürtel 18-20, 1090 Vienna, Austria; 2 Institute of Materials Science and Technology, Faculty of Mechanical and Industrial Engineering, TU Wien, Getreidemarkt 9, 1060 Vienna, Austria; 3 Center for Anatomy and Cell Biology, Medical University of Vienna, Währinger Straße 13, 1090 Vienna, Austria

**Keywords:** hand biomechanics, pulley reconstruction, pulley injury, climbing injury, tenodesis screws, PL graft

## Abstract

**Background**: The aim of this biomechanical investigation was to evaluate a V-shaped three-point graft tenodesis technique using a free palmaris longus (PL) tendon for reconstructing traumatic A2 pulley lesions and to compare its biomechanical performance with two previously described reconstruction techniques. **Methods**: After A2 pulley lesion simulation in 27 fingers (index, middle and ring finger) from nine human anatomical hand specimens, reconstructions were performed using the innovative V-shaped graft tenodesis technique, a double-loop encircling technique and a suture anchor graft fixation technique. Load at failure and the failure mechanisms were evaluated. **Results**: The V-shaped graft tenodesis technique was superior biomechanically (*p* = 0.004) considering load at failure (mean: 299 N). The only observed failure mechanism in this group was the extrusion of the central tenodesis screw. In contrast, reconstructions in the other two groups failed due to suture cut-out. **Conclusions**: Patients may benefit from the new technique’s high load tolerance during early mobilization. Furthermore, a reduction in complications may be anticipated due to an absence of sutures and the sparing of extensor structures.

## 1. Introduction

The pulley system within the flexor tendon sheath of the hand is essential for preserving the sheath’s structural integrity and maintaining the dynamic relationship between the flexor tendon and the bony structures of the finger during flexion and extension [1,2,3,4]. Closed injury to this complex arrangement of ligamentous structures may not only compromise finger function in the acute phase due to pain and swelling but, if left untreated, may lead to bowstringing accompanied by a loss of strength and a fixed flexion contracture at the proximal interphalangeal (PIP) joint [5,6,7,8,9,10,11]. Due to its anatomy, location and microstructure, the A2 pulley was proven to be the biomechanically most relevant structure of the flexor tendon sheath, as an untreated injury to this tendon-restraining ligament bears the highest risk of inducing clinically relevant limitations to finger and hand function. In rock climbers, this injury prevalently occurs in the crimp grip position [11,12]. Unlike other hand positions (e.g., slope grip), the crimp grip combines flexion in the metacarpophalangeal (MCP) joint and the PIP joint, accompanied by a hyperextension of the distal interphalangeal (DIP) joint. Due to the increased moment arm of the flexor tendons over the PIP joint during flexion, this configuration amplifies the forces transmitted to the A2 pulley, making it particularly prone to rupture. Consequently, the crimp grip seems to be the most clinically and biomechanically relevant finger posture for evaluating pulley failure and reconstruction techniques [12,13,14,15].

The therapeutic approach varies from conservative treatment to complex pulley reconstruction procedures depending on the number of affected pulleys, accompanying injuries, demands of the patient, and delays in diagnosis [16]. Over the last two decades, nonsurgical treatment has become the standard therapy for closed single-pulley injuries [17,18,19,20,21,22,23,24,25,26]. In more complex injury patterns, or persistent impairment of finger function due to gross pulley insufficiency or complete rupture, a variety of reconstruction techniques have been described, and some of these have found their way into everyday surgical practice [4,17,27,28,29,30,31,32,33,34,35,36,37,38,39,40,41,42]. When utilizing a tendon graft for pulley reconstruction, loop techniques or non-encircling techniques can be used depending on the surgeon’s preference, the quality of the A2 pulley remnants and the functional demands of the patient. In the majority of applied techniques, reconstructions tend to fail either at the graft suture site due to suture cut-out or at the interface between the graft and the remaining rims of the ruptured pulley remnants, which are used to secure the graft [34,40]. Mallo et al. [30] compared three reconstruction techniques to native A2 pulleys in a biomechanical study using a setup focusing on a direct pull-off mechanism. He reported mean failure loads of 181.5 N for native A2 pulleys, while the loads were 165.4 N for the double-loop technique and 65.9 N for the double anchor technique. Despite their previously described biomechanical advantages, evidence suggests that loop techniques may not only irritate the extensor structures, leading to an extensor block, but may also result in potential cortical bone loss of the proximal phalanx due to interference with its cortical blood supply [21,34,43]. Additionally, significant adhesion formation can be promoted by excessive use of suture material in loop techniques and suture anchor graft fixations, as well as by a limited ability for early mobilization due to the lower load tolerance of certain reconstruction techniques [44,45,46].

The concept of transosseous graft placement for pulley reconstruction has been previously studied and published by Schöffl et al. [40], demonstrating no tendency towards bone weakening and subsequent proximal phalanx fracture when loaded to failure. In their study, the reconstruction primarily failed at the distal end of the tendon graft, where it was threaded through the remaining rims of the previous pulley, while the transosseous portion of the reconstruction remained intact in all tested specimens. The mean recorded failure loads measured at the flexor digitorum profundus (FDP) tendon were 212.4 N in the reconstruction group compared to 292.4 N in native pulleys.

The development of small-sized tenodesis screws allows for intraosseous free tendon graft fixation within the proximal phalanx. This led us to propose an alternative approach to A2 pulley reconstruction, aiming to mitigate unintended postoperative effects such as extensor tendon irritation, cortical bone loss or insufficiency due to suture cut-out. Tenodesis-based techniques, by reducing reliance on sutures and enabling early mobilization through increased reconstruction strength, may have the potential to decrease adhesion-related complications, improve functional recovery, and reduce the need for revision surgeries. If these benefits are present, they may justify the higher initial costs associated with techniques that are dependent on implants.

This study aims to biomechanically evaluate a V-shaped three-point intraosseous graft fixation, hypothesizing that it provides sufficient rigidity and stability when subjected to failure loads while simulating a crimp grip position. To highlight the advantages of the innovative reconstruction technique, we compared its biomechanical failure characteristics to those of a double-loop encircling technique and a graft fixation using two suture anchors.

## 2. Materials and Methods

A range of biomechanical testing protocols for evaluating pulley strength after repair or reconstruction were identified in the literature [9,30,34,41,42,47,48,49]. Some of these protocols appeared to more accurately simulate physiological loading conditions than others. Concordant to biomechanical evaluations of flexor tendon injuries with a preserved flexor tendon sheath, we embraced a curvilinear testing model to examine biomechanical characteristics when repairs are loaded to failure. All specimens were obtained at the Center for Anatomy and Cell Biology, Medical University of Vienna, where all of the body donors provided written informed consent prior to their death for use in teaching and science. The Institutional Ethics Committee of the Medical University of Vienna approved this study (ID number: 1436/2019).

### 2.1. Validation of the Biomechanical Setup

Prior to evaluating reconstruction techniques, we conducted a series of load to failure trials on native A2 pulleys to validate our biomechanical setup. The purpose of those trials was to confirm that the bespoke test fixture (detailed in Section 2.3) could reliably replicate and maintain the crimp grip position throughout testing and that we were able to accurately simulate the A2 pulley injury mechanism. Additionally, we evaluated whether the failure loads observed in native A2 pulleys using our setup aligned with previously reported values in the literature. The trials were performed under identical conditions to those used for testing reconstructed pulleys. Nine fingers from three human hand specimens were prepared, mounted on the test fixture, and loaded to failure according to the standard protocol described below. The results demonstrated consistent replication of the injury mechanism: the crimp grip was successfully maintained throughout testing, and no extraneous events, such as fractures, tendon extrusions or fixture failures, occurred. In all trials, the failure occurred exclusively as a rupture of the A2 pulley with no damage to surrounding structures or fixation points. The mean load at failure value of native A2 pulleys in the crimp grip position was 375 ± 70 N. Our result aligns closely with the calculated force of 450 N by Bollen et al. [5], the reported maximum strength of the A2 pulley at 407 N by Lin et al. [50] and the 375 N failure load described by Tang et al. [51]. This validation process confirmed the reliability and accuracy of our biomechanical setup, making it suitable for subsequent testing of reconstruction techniques.

### 2.2. Specimen Dissection and Pulley Reconstruction

Nine fresh-frozen hand specimens (four female, five male) were randomly selected (mean 75, range 65–85 years of age). According to our standard protocol in human specimen preparation, the specimens were thawed at 4 °C for 48 h prior to injury simulation and pulley reconstruction. Donor records were reviewed for particular diseases, leading to loss of bone density. During dissection of the specimens, no signs of impaired bone quality, previous surgeries, or flexor tendon sheath injuries were recognized. Preparation of the nine hand specimens for the biomechanical testing began with the transection of radius and ulna 10 cm proximal to the radiocarpal joint. At their musculotendinous junction, all flexor tendons were carefully separated from muscular tissue, and their anatomical affiliations were identified. The tendons of the flexor carpi radialis (FCR), flexor carpi ulnaris (FCU) and flexor pollicis longus (FPL), as well as the flexor digitorum superficialis (FDS) and flexor digitorum profundus (FDP) tendon of the small finger, were transected at the level of the radiocarpal joint. The palmaris longus (PL) tendon was harvested in toto and kept in a physiological saline solution until grafting. The FDS and FDP tendons of each index, middle and ring finger were separated from their intertendinous connections to enable selective flexion of each finger. The flexor tendons of each finger were interlaced using a Pulvertaft technique and secured with an interlocking suture employing a #5 FibreWire (Arthrex, Naples, FL, USA) suture. The creation of this tendon loop provided an equal distribution of tension forces on the flexor tendon sheath when placed around a deflection pulley mounted on the tensile testing machine, simulating traction of both flexor muscles equally. Hereafter, careful dissection of the flexor tendon sheath was performed in the index, middle and ring fingers. In these fingers, the volar and lateral portion of the skin was excised from the level of the transverse palmar crease to the distal interphalangeal (DIP) joint, thereby exposing the flexor tendon sheath. Pulleys A1 to A4 were visualized and cleared of adherent subcutaneous tissues while preserving interligamentary structures. The A2 pulleys were identified, and their lengths were measured in millimeters. Subsequently, the A2 pulleys were resected at their osseous insertions on the proximal phalanx. This concluded the specimen preparation and injury simulation. Rehydration of the specimen was ensured by storage in saline solution (0.9% NaCl) until testing. Throughout the dissection, specimens were irrigated with this solution periodically.

**Figure 1:**
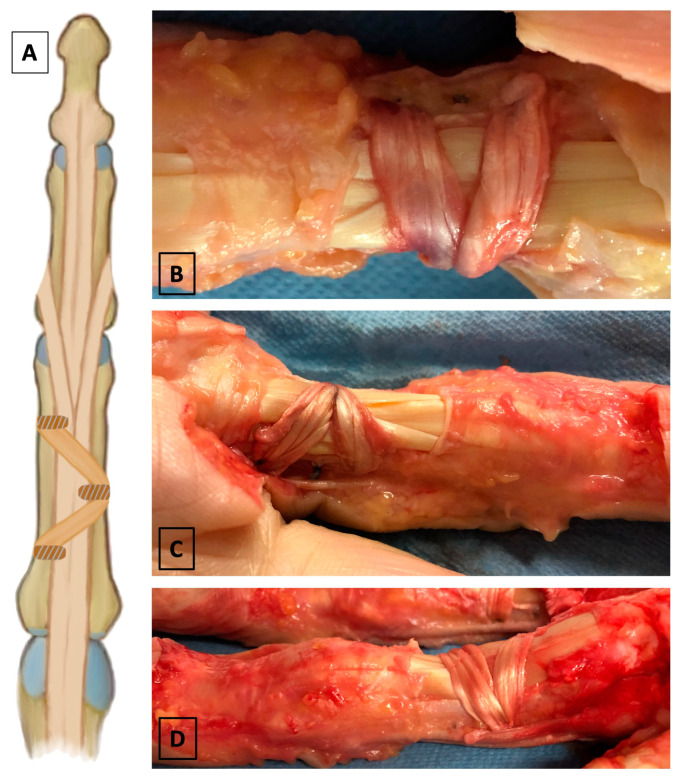
(**A**) Illustration of the V-shaped graft tenodesis technique. (**B**) Top view of a specimen following A2 pulley reconstruction using this technique. (**C**,**D**) Oblique views of the reconstruction depicting graft position as well as the pattern of tenodesis screw placement within the proximal phalanx.

Three study groups were established through random allocation, with each group consisting of three hands, resulting in a total of nine finger specimens per group. In the first group (termed “tenodesis”), reconstruction was conducted using a 10 cm PL tendon graft. The graft was inserted into the proximal phalanx in a V-shaped three-point configuration and secured with three 2.5 mm × 6 mm PEEK tenodesis screws (Arthrex, Naples, FL, USA). This technique follows the idea of avoiding suture material to be the sole graft-capturing material, therefore counteracting the potential complication of suture cut-out. Two parallel guidewires were drilled bicortically into the midcoronal aspect of the proximal phalanx and positioned at the distal and proximal margins of the former A2 pulley. A third guidewire was then bicortically drilled from the contralateral side at the estimated center of the former A2 pulley across the proximal phalanx. Taking into account the vector of flexor tendon force, which pulls toward the center of the palm and subsequently into the carpal tunnel, causing increased stress on the corresponding sections of the A1 and the A2 pulley, the two ipsilateral guidewires were introduced from the ulnar side in the index and the middle fingers and from the radial side in the ring finger, respectively [52]. Both parallel guidewires were overdrilled with a 2 mm cannulated drill to create a monocortical blind hole. On the contralateral side, a 2.5 mm cannulated drill was used to create a slightly larger monocortical blind hole to accommodate the PL tendon graft loop. A #2-0 FibreWire (Arthrex, Naples, FL, USA) was woven into both ends of the tendon graft using a whipstitch suture pattern. A loop of FibreWire thread was placed around the graft. The sutures securing the graft ends were passed into the two parallel blind holes on one side of the proximal phalanx using eyelet guidewires. The FibreWire loop was then passed through the larger central blind hole, facilitating the insertion of the graft loop into the proximal phalanx. After confirming the desired graft tension and free gliding of the flexor tendons, the tenodesis screws were inserted into all three blind holes and firmly tightened. Excess suture material was trimmed flush with the corresponding contralateral cortices (Figure 1).

**Figure 2:**
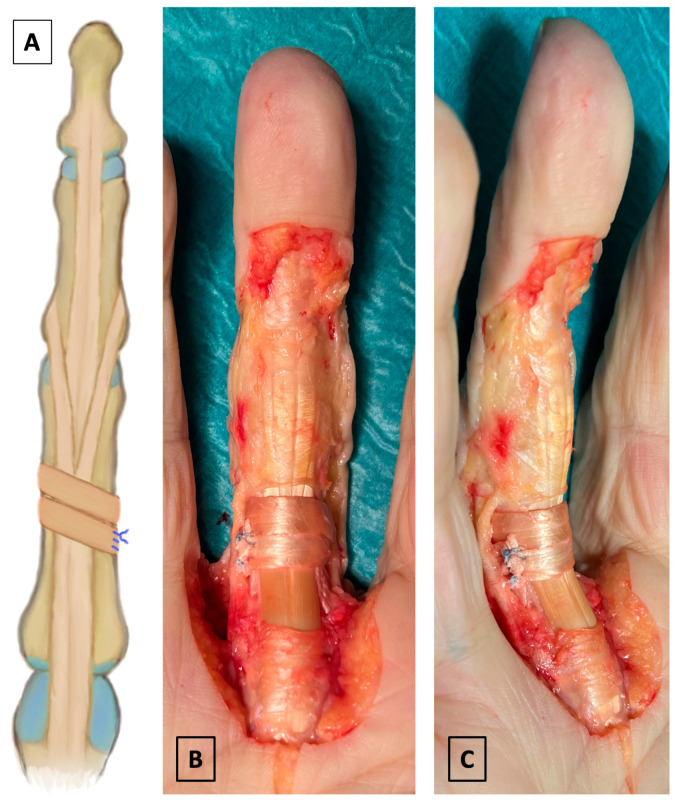
(**A**) Illustration of the double-loop technique. (**B**,**C**) Top and oblique views of the double-loop technique.

We utilized a double-loop technique for the reconstruction of the A2 pulley in the second group (termed “loop”) [30,33,53]. At the site of the resected A2 pulley, a free PL tendon graft was threaded circumferentially around the flexor tendons and underneath the retracted extensor tendons, passing through twice. When assessing the tension of the reconstruction, particular attention was given to ensuring unobstructed gliding of the flexor tendons. The graft ends were secured with an uninterrupted suture using #2-0 FiberWire (Arthrex, Naples, FL, USA) (Figure 2).

**Figure 3:**
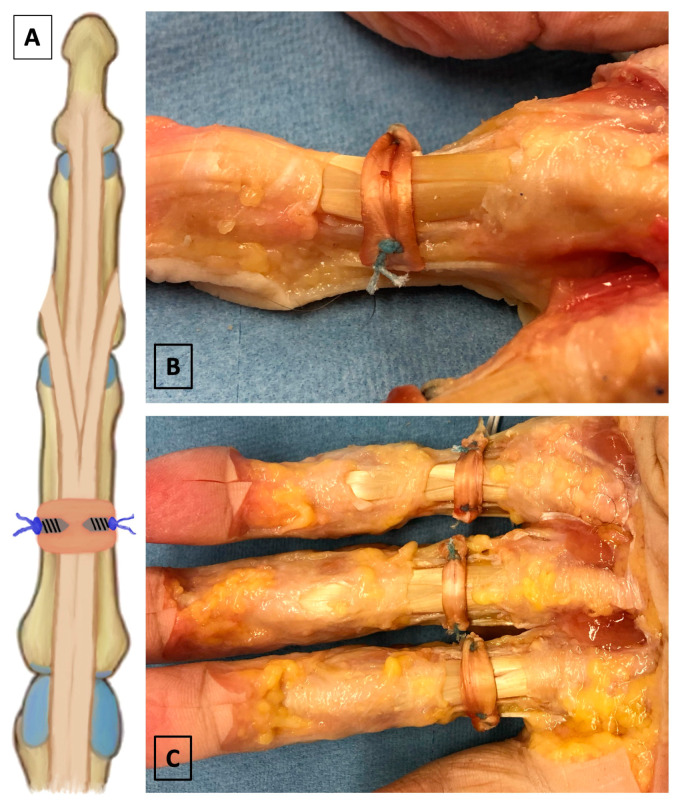
(**A**) Illustration of the anchor technique. Positioning of the suture anchors and the graft are visualized. (**B**,**C**) Oblique and top views of the anchor fixation of the PL tendon graft.

For the reconstruction of the A2 pulley in the third group (termed “anchor”), we used two suture anchors for the fixation of the free tendon graft [30]. At the center of the bony attachment of the former A2 pulley, one Micro Corkscrew FT, 2.2 mm × 4.0 mm suture anchor loaded with #2-0 FiberWire sutures (Arthrex, Naples, FL, USA) was inserted on the radial aspect, while another one was inserted on the ulnar aspect of the proximal phalanx. The PL tendon graft was tied to the bone on each side with a horizontal mattress suture and secured with a 5 throw square knot (Figure 3).

### 2.3. Data Acquisition and Testing

**Figure 4:**
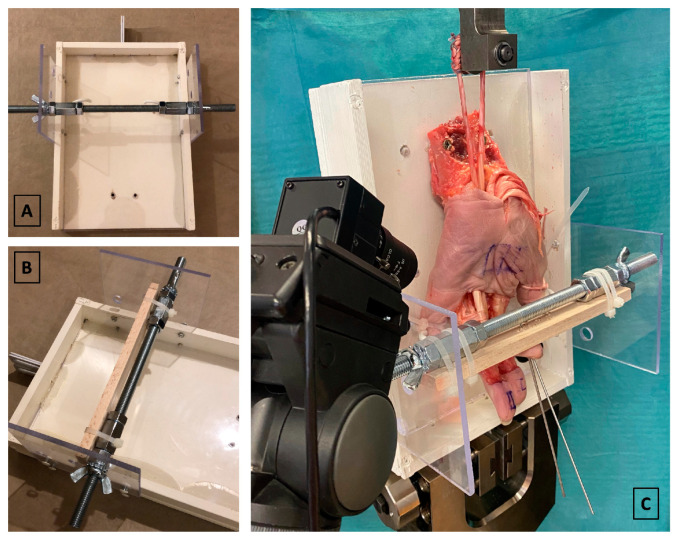
(**A**) Top view of the test fixture. (**B**) Oblique view of the test fixture. (**C**) Test fixture with the prepared specimen mounted on the tensile testing machine.

The design of a bespoke test fixture allowed the hand specimens to be held securely, facilitated the simulation of the desired grip position and enabled visualization of the dynamics of the A2 pulley reconstruction throughout biomechanical testing. The test fixture was constructed from a rectangular wooden tray lined with plexiglas. In addition, two symmetrically arranged plexiglas plates were attached to the long sides of the tray. Three holes in each of the two side plates allowed for accommodation of a metallic rod with a wooden bar attached for positioning the finger being tested in the desired grip position. To secure the specimen to the base plate of the tray, holes were drilled into the radius and ulna, and each bone was fixed to the tray with a screw. Additionally, the thumb and small finger were held to the tray by two metallic hooks. The finger to be examined was positioned in the test fixture to simulate a “crimp grip” with an angulation of 30° at the MCP joint, 90° to 100° in the PIP joint, and 5° of hyperextension in the DIP joint. The distal phalanx was secured to the transverse wooden bar with two 2 mm K-wires. The test fixture, with the prepared specimen, was mounted on an electromechanical tensile testing machine (Zwick Z050, ZwickRoell GmbH, Ulm, Germany). The FDS and FDP tendon loop was placed around a deflection pulley which was equipped with a 2.5 kN load cell. Prior to testing, the load cell was checked for its accuracy in the low load region using calibrated weights. In the range from 0.1 to 5 N the maximum deviation was 7 mN. A camera was placed at a 45° angle to the tray plane to record the failure characteristics and failure mechanisms throughout testing (Figure 4). A preload of 2 N was applied to the interlaced flexor tendon loop. The trial was initiated, and the tendons were continuously loaded at a rate of 100 mm/min. At this point, a displacement versus force curve was generated and the video recording started automatically. A sudden and steep drop in load of 50 N indicated either rupture of the pulley reconstruction or any other event of failure. This terminated the trial, stopped the tensile testing machine and halted the video recording. By reviewing the force versus displacement curve, the load at failure of the pulley reconstruction was determined. The failure mechanism and any potential accompanying injuries were documented through video analysis and subsequent examination of the specimen.

### 2.4. Statistical Analysis

A statistical analysis was performed using IBM SPSS Software, Version 26, 64-bit. Descriptive statistics, including means, standard deviations, and ranges, were calculated for each group. The normality of the data was assessed using the Shapiro–Wilk Test. For the load at failure parameter, the loop group did not meet the assumption of normality. Subsequently, a Kruskal–Wallis one-way analysis of variance was performed to assess differences among the three repair techniques. For post hoc analysis, a Dunn’s test with a Bonferroni correction was applied for pairwise comparison among the groups. Given the small range and limited variability in the pulley length values, the Kruskal–Wallis test was also applied to compare pulley lengths between groups. *p*-values below 0.05 were considered statistically significant before correction, and adjusted *p*-values were reported for pairwise comparisons within the load at failure parameter.

## 3. Results

**Figure 5:**
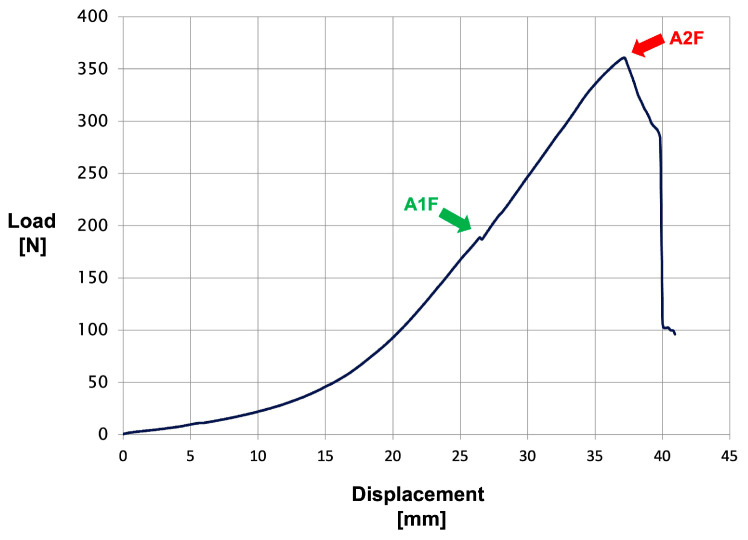
Exemplary load versus displacement curve in the tenodesis group. The arrow labeled “A1F” marks the failure point of the A1 pulley, while the arrow labeled “A2F” marks the load at failure of the A2 pulley.

During the reconstructions, no adverse events arose from the procedures or specimen quality. The biomechanical setup met our expectations, providing a rigid hold of the hand, proper positioning of the tested finger, equal strain distribution to both flexor tendons, and firm capture of the tendon loop. We were able to maintain the crimp grip position throughout the testing and simulate an A2 pulley reconstruction failure in each specimen in a consistent manner. To outline the homogeneity of our specimens, we measured the native pulley length. We observed no statistically significant difference in this parameter between our study groups (*p* = 0.32). The mean length of the A2 pulleys was 17.1 ± 2.2 mm in the tenodesis group, 15.8 ± 1.7 mm in the loop group and 16.3 ± 2.5 mm in the anchor group. When loaded to failure, no particular accompanying injuries occurred, such as fracture of the phalanges or metacarpals, flexor tendon sheath ruptures distal to the A2 pulley, tear or avulsion of the flexor tendons or extrusion of the finger from its fixation on the wooden bar or premature implant tear-out; we also did not encounter any technical failure of the testing machine. When the applied load reached its critical magnitude, a rupture of the A1 pulley was frequently present prior to failure of the A2 pulley reconstruction (Figure 5).

**Figure 6:**
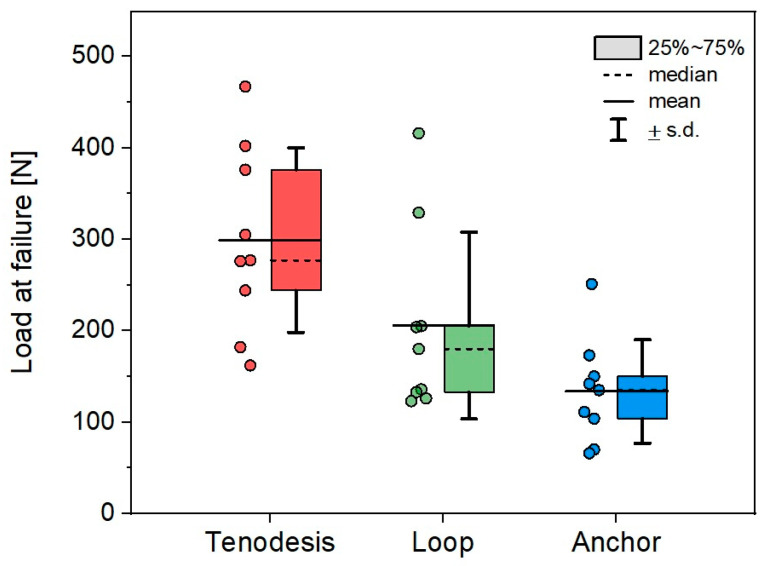
Boxplot illustrating the distribution of load at failure values across the three groups—tenodesis, loop and anchor. Colored boxplots were used to differentiate the three study groups, and individual data points were included to visualize sample variability and distribution.

In the tenodesis group, we observed a mean load at failure of 299 ± 100.8 N, compared to 205.7 ± 102.1 N in the loop group and 133.5 ± 56.6 N in the anchor group (Figure 6). The Kruskal–Wallis test showed an overall statistically significant difference in load at failure among the three study groups (*p* = 0.004), indicating the potential superiority of the proposed V-shaped three-point graft tenodesis technique in regard to load tolerance. Nevertheless, no statistical significance of load at failure values between the tenodesis and loop group (*p* = 0.21), as well as the loop and anchor group (*p* = 0.36), could be identified. Regarding the failure mechanism, in the tenodesis group we consistently observed an extrusion of the palmaris longus tendon graft along with the tenodesis screw from the central blind hole. The two ipsilateral tenodesis screws showed no tendency towards extrusion, even upon meticulous examination following each trial. The graft itself remained intact, with no signs of laceration. The bone surrounding the blind holes, particularly the central blind hole, displayed no osseous avulsion marks. There were no visible signs of fracture lines across the entire proximal phalanx. In the reconstruction groups using the double-loop encircling technique and suture anchor fixation of the graft, the sutures consistently cut through the tendon graft as the failure mechanism. In the anchor group, the suture-graft interface failure occurred without a distinct side being preferentially affected. We did not observe any suture anchor extrusion from the bone.

## 4. Discussion

The aim of this study was to investigate the biomechanical characteristics of a novel V-shaped three-point intraosseous graft fixation using tenodesis screws for an A2 pulley reconstruction and to compare this method with two established surgical techniques. The key finding of our investigation was the superior biomechanical performance of the proposed technique, demonstrating a higher load tolerance when subjected to failure loads. When comparing our biomechanical test fixture with previously published biomechanical testing setups, it seems important to note that none of our trials had to be discarded due to specimen damage caused by the setup itself [40,48]. Furthermore, a comprehensive review of the diverse biomechanical testing setups and specimen fixtures used in previous studies revealed a lack of a physiological reproduction of the injury pattern in some cases. Specifically, in some studies, strain was applied exclusively to the A2 pulley for a pull-off test using a metallic hook or a tendon slip inserted into the A2 pulley space following flexor tendon resection [3,30,34,41,47,50]. In response to these findings, we chose a functional loading test utilizing a “whole-hand” specimen test fixture, inspired by the methodology of Warme et al. [48], enabling a more physiological replication of the injury pattern.

Native A2 pulleys withstand loads of up to 400 N [50,54]. According to Mallo et al. [30], the failure load of the double-loop technique was closer to that of the native A2 pulley compared to the anchor technique. In addition to our innovative graft tenodesis technique demonstrating failure loads closest to those of a native A2 pulley, our findings are consistent with previous studies showing that the double-loop technique performs better than the suture anchor technique.

Although Schöffl et al. [40] successfully proposed a technique using a transosseous placement of the tendon graft, a three-point intraosseous fixation raised some concerns about bone weakening. However, another positive observation in our study was the absence of a proximal phalanx fracture across all biomechanical trials. The sole mechanism of failure in the tenodesis group was the extrusion of the central tenodesis screw. We believe that the reason is an equal distribution of tension forces along the tendon graft loop. This likely created a consistent extrusion force that pulled the screw out of the blind hole. Furthermore, the two tendon slips situated on the outer surface of the screw may have reduced the contact area and thereby lowered the central screw’s hold within the bone.

Reconstructions in the loop and anchor groups solely failed due to suture cut-out. As presented by Slesarenko et al. [41] and Mallo et al. [30], the suture anchor reconstruction allowed a minimally invasive approach on one hand but displayed a low load tolerance compared to other techniques on the other hand. Although biomechanically superior to suture anchor-based reconstructions, the double-loop technique still depends on a secure suture capture of the graft. Under excessive repetitive loading or a single high-load event, the sutures may cut through the graft, leading to a failure of the reconstruction.

Due to the re-popularization of sports such as rock-climbing and bouldering, the incidence of pulley injuries has significantly increased in recent years [16,21,55,56,57]. Bouldering, in particular, places unique demands on the A2 pulley due to its high-intensity, short-duration routes that often require maximal grip strength combined with explosive dynamic loading of flexor tendons and their surrounding structures. This results in extreme forces being transmitted through the pulley system, approaching or even exceeding failure loads observed in native A2 pulleys [15]. Holds with a small surface area may increase the risk of pulley injury by necessitating frequent use of more stressful hand positions, such as the crimp grip. These factors underscore the importance of reconstruction techniques with a high load tolerance. The novel V-shaped tenodesis technique evaluated in this study demonstrates failure loads that may provide adequate strength to meet the functional requirements of high-demand athletes. Its enhanced mechanical stability and sutureless design could allow for earlier mobilization, potentially reducing adhesion formation and promoting the restoration of a satisfactory grip strength. This approach may enable patients to achieve a safer and faster return to work and sports.

There are limitations to this study. While appropriate for an ex vivo biomechanical investigation, the use of cadaveric specimens cannot fully replicate the changes in biomechanical properties throughout the healing process. Furthermore, we are not able to predict the effects of repetitive loading on the reconstructed pulley. Additionally, our setup does not account for scar tissue formation, which may increase the risk of suture cut-out during the inflammatory phase of the healing process. Excessive adhesion formation could further alter load distribution on the reconstructed pulley, increasing the risk of delayed reconstruction insufficiency or even failure. The age of the specimens used in this study may also represent a limitation, as bone quality and tendon elasticity can vary between younger and older populations, potentially influencing outcomes in a biomechanical study. The small sample size of nine specimens per group, while providing meaningful data, may also be considered a limitation. The biomechanical testing was performed in a crimp grip position, being relevant to climbers, but may not reflect the whole variety of hand positioning and loading conditions experienced by all patients suffering from A2 pulley injuries. To evaluate the graft-bone integration using this particular technique, and to understand the tendency towards potential complications that cannot be anticipated in an ex vivo biomechanical study, subsequent clinical trials will be essential.

## 5. Conclusions

In this biomechanical study on a V-shaped three-point A2 pulley reconstruction technique utilizing an intraosseous PL tendon graft fixation with tenodesis screws, we were able to underline its superior biomechanical performance compared to traditional encircling and suture anchor-based techniques. Our findings indicate a promising alternative for surgeons treating patients with complex pulley injuries, particularly high-demand athletes and manual workers. However, future clinical investigations will assess the technique’s characteristics in vivo throughout the healing process, thus revealing its relevance in surgical practice.

## Data Availability

The data that support the findings of this study are available upon reasonable request from the corresponding author [G.H.].
